# Glomerular Filtration Rate and Proteinuria: Association with Mortality and Renal Progression in a Prospective Cohort of a Community-Based Elderly Population

**DOI:** 10.1371/journal.pone.0094120

**Published:** 2014-04-07

**Authors:** Se Won Oh, Sejoong Kim, Ki Young Na, Ki Woong Kim, Dong-Wan Chae, Ho Jun Chin

**Affiliations:** 1 Department of Internal Medicine, Inje University College of Medicine, Goyang City, Gyeonggi-do, Korea; 2 Department of Internal Medicine, Seoul National University Bundang Hopsital, Seong-Nam, Korea; 3 Department of Internal Medicine, Seoul National University College of Medicine, Seoul, Korea; 4 Department of Psychiatry, Seoul National University College of Medicine, Seoul, Korea; UNIFESP Federal University of São Paulo, Brazil

## Abstract

Limited prospective data are available on the importance of estimated glomerular filtration rate (GFR) and proteinuria in the prediction of all-cause mortality (ACM) in community-based elderly populations. We examined the relationship between GFR or proteinuria and ACM in 949 randomly selected community-dwelling elderly subjects (aged ≥65 years) over a 5-year period. A spot urine sample was used to measure proteinuria by the dipstick test, and GFR was estimated using the chronic kidney disease-epidemiology collaboration (CKD-EPI) equation. Information about mortality and causes of death was collected by direct enquiry with the subjects and from the national mortality data. Compared to subjects without proteinuria, those with proteinuria of grade ≥1+ had a 1.725-fold (1.134–2.625) higher risk of ACM. Compared to subjects with GFR ≥90 ml/min/1.73 m^2^, those with GFR<45 ml/min/1.73 m^2^ had a 2.357 -fold (1.170–4.750) higher risk for ACM. Among the 403 subjects included in the analysis of renal progression, the annual rate of GFR change during follow-up period was −0.52±2.35 ml/min/1.73 m^2^/year. The renal progression rate was 7.315-fold (1.841–29.071) higher in subjects with GFR<60 ml/min/1.73 m^2^ than in those with GFR ≥60 ml/min/1.73 m^2^. Among a community-dwelling elderly Korean population, decreased GFR of <45 ml/min/1.73 m^2^ and proteinuria were independent risk factors for ACM.

## Introduction

The prevalence of chronic kidney disease (CKD) has been increasing, causing concern both worldwide and in Korea [1]. Age is one of the most important risk factors related to the prevalence of CKD. The prevalence of a glomerular filtration rate (GFR) of <60 ml/min/1.73 m^2^ is 0.9% in subjects aged <60 years but increases to 51.2% among those aged ≥80 years in the USA [2]. The prevalence of albuminuria has been reported as 32.7% among a population of subjects aged ≥80 years [2]. However, studies evaluating the association of age with incident CKD among community-dwelling elderly populations in a prospective cohort are limited [2]. Studies on GFR changes with aging have shown that GFR declines steadily at a rate of 0.96 ml/min/1.73 m^2^ annually after 30–40 years of age and decreased more rapidly after the age of 60 years [3], [4]. Therefore, it is debatable whether mildly decreased GFR without definite evidences of renal damage, such as proteinuria or azotemia-related complications, should be considered as a “disease” in the elderly population [5]. In older populations, the prevalence of some of the CKD-related complications in subjects with GFR of 45–59 ml/min/1.73 m^2^ was not higher than that in subjects with GFR ≥60 ml/min/1.73 m^2^ [6]. A recent meta-analysis by the CKD prognosis consortium showed that the risks for mortality and renal progression were high in older populations with mildly decreased GFR [7]. However, there were several points to be cleared, which were reported in community-based prospective cohort studies. GFR has not been found to be a risk factor for all-cause mortality (ACM) [8], and the criterion of decreased renal function, in terms of GFR<60 ml/min/1.73 m^2^, has been found to be less suitable for the prediction of cardiovascular mortality (CVM) [9] and ACM [10]. Furthermore, albuminuria, rather than GFR, has been found to be a risk factor for incident cardiovascular disease [11]. Another issue is that Korean data included in the consortium were obtained from participants who underwent health examination in 2 selected centers and did not represent the community population [12]. Therefore, we sought to prospectively analyze the decline in the GFR, incidence of renal progression, and the effect of GFR and proteinuria on ACM in a randomly selected, community-based, elderly population residing in a Korean city, over a 5-year follow-up period.

## Materials and Methods

### Design of KLoSHA and Study Population

**Figure 1:**
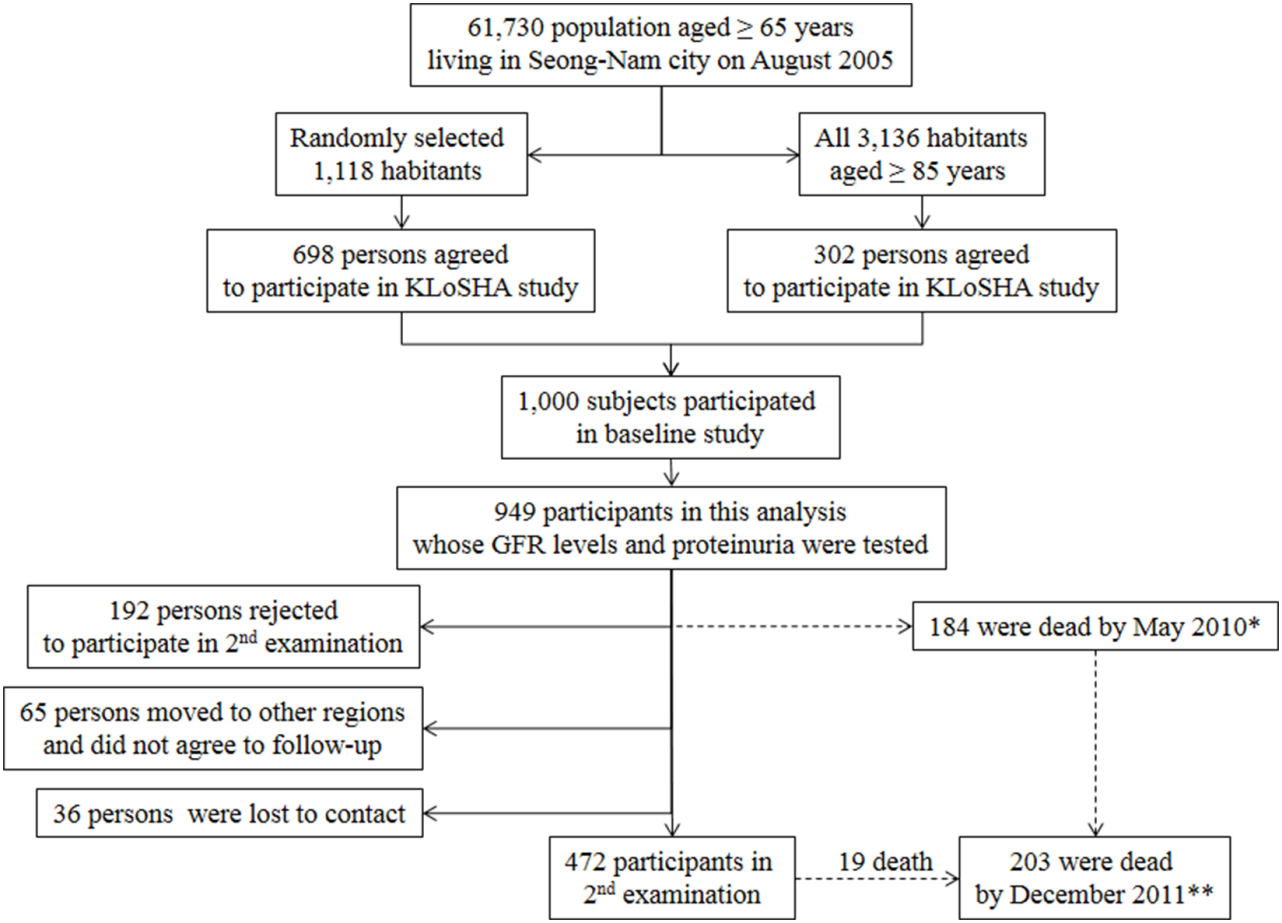
Participants in KLoSHA study. *Mortality was detected by direct contact and the national database. **Mortality was identified by the national database.

This study was conducted as a part of the Korean Longitudinal Study on Health and Aging (KLoSHA), which included a randomly selected, community-based, elderly population. The detailed design of the KLoSHA has been described in our previous report [13]. This study protocol was reviewed and approved by the institutional review board of the Seoul National University Bundang Hospital (B-0508/023-003). The study was conducted in accordance with the Declaration of Helsinki. The baseline study was conducted from September 2005 to September 2006; the follow-up study, from May 2010 to March 2012. After obtaining written informed consent from all participants, the assessments were performed at SNUBH. Among 1,000 subjects originally included in the KLoSHA, 949 subjects with baseline serum creatinine and proteinuria, as determined by the dipstick test (Figure 1) were enrolled in the current study.

### Demographic and Clinical Characteristics

Blood pressure values considered for the analysis were the means of 3 measurements. All medications taken by the participants were gathered and identified. Diabetes mellitus (DM) was defined by the use of anti-diabetic medicines, a serum fasting glucose level ≥126 mg/dl, or hemoglobin A1c (HbA1c) level ≥6.5%. Hypertension was defined as systolic blood pressure (SBP) ≥140 mmHg, diastolic blood pressure (DBP) ≥90 mmHg, or the use of anti-hypertensive medication. Proteinuria was defined as protein ≥1+, as determined by a dipstick urine test, while hematuria was defined as a red blood cell (RBC) count ≥5 per high-power field, as examined by a light microscopic examination of a urine sample. Serum creatinine level was measured by the alkaline picrate Jaffe kinetic method using an automatic analyzer (Toshiba 200FR, Tokyo, Japan). Serum creatinine levels were calibrated to an assay traceable on an isotope dilution mass spectrometry (IDMS) device (Roche diagnostics). GFR was calculated using the CKD-epidemiology collaboration (CKD-EPI) equation [14]. The participants were categorized into 4 groups on the basis of the GFR. Proteinuria was determined by the urine dipstick test, using an automated urine analyzer (CLINITEK ATLAS, Siemens Healthcare Diagnostics, Deerfield, IL, USA). Anemia was defined by hemoglobin levels <12 g/dL and <13 g/dL in women and men, respectively.

### Outcomes

We obtained information about the survival status of the subjects participating in the baseline study via contact through telephone, cellular phone, and/or mail. This information was then confirmed by comparison against the mortality data maintained by the National Statistical Office (NSO) of Korea for the period between September 2005 and December 2011, using each individual’s unique identification number. The estimated GFR decline rate for the participants who were examined a second time was determined using covariate analysis (ANCOVA). We defined renal progression as an annual decrease rate in GFR of ≥2.5 ml/min/1.73 m^2^/year during the follow-up period and GFR<45 ml/min/1.73 m^2^ at the second examination [15].

### Statistical Analyses

All analyses were performed using SPSS (SPSS version 20.0, Chicago, IL). We compared the cumulative incidences of ACM among the participants by using the log-rank test. Cox’s hazard proportional analysis was used to estimate the hazard ratios (HRs) for mortality. HRs for ACM was adjusted for age, gender, habit of smoking, CRP, cholesterol, triglyceride, albumin, platelet, and hemoglobin. We selected these adjusting factors which showed significant associations with mortality in the univariate analysis. Formal tests for multiplicative interactions were conducted by comparing −2 log likelihood in regression models, including the full population, with and without interaction terms (for example, risk factor 1 * risk factor 2). We compared the incidence of renal progression in the different groups using logistic regression analysis. The number of subjects with renal progression was small, and we grouped the subjects according to the GFR level of 60 ml/min/1.73 m^2^. P-values of 0.05 were considered statistically significant.

## Results

### Baseline Characteristics

The mean age of the participants was 75.8±9.0 years. DM was present in 24.2% of the subjects; hypertension, in 73.8%. The GFR was ≥90 ml/min/1.73 m^2^ in 15.2% of the subjects; 60–89 ml/min/1.73 m^2^, in 60.5%; 45–59 ml/min/1.73 m^2^, in 16.5%; and <45 ml/min/1.73 m^2^, in 7.8%. Proteinuria of grade ≥1+was noted in 8.2% of the population. The proportion of subjects with GFR<60 ml/min/1.73 m^2^ was higher in the oldest old group, aged ≥75 years, than in the group of subjects aged 65–74 years (41.2% vs. 11.2%) (Table 1).

**Table 1:** Basal characteristics of elderly population at baseline study according to age.

	All	Age<75 years	Age≥75 years	p-value
Number	949	534	415	
Age (years)	75.8±9.0	68.8±2.9	84.8±5.6	<0.001
Gender (Male, %)	45.4	45.5	45.3	0.950
BMI (kg/m^2^)	24.0±3.3	24.6±3.2	22.9±3.2	<0.001
DBP (mmHg)	82.7±10.6	83.5±10.3	81.5±10.9	0.003
SBP (mmHg)	132.3±17.9	132.3±16.8	132.3±19.2	0.973
Smoking (%)				0.202
Never	58.9	59.9	57.6	
Ex-smoker	29.6	27.5	32.3	
Current smoker	11.5	12.5	10.1	
Diabetes mellitus (%)	24.2	25.7	22.4	0.328
Hypertension (%)	73.8	72.6	75.4	0.279
Hemoglobin (g/dL)	13.7±1.5	14.0±1.4	13.3±1.5	<0.001
Anemia (%)	8.6	4.3	14.3	<0.001
Creatinine (mg/dL)	0.93±0.37	0.89±0.35	0.98±0.40	0.001
GFR (ml/min/1.73 m^2^)	72.2±17.0	78.1±14.5	64.6±17.0	<0.001
≥90	15.2	24.2	3.6	<0.001
60–89	60.5	64.6	55.2	
45–59	16.5	8.2	27.2	
<45	7.8	3.0	14.0	
Proteinuria by dipstick (%)				0.012
none	83.6	86.7	79.5	
trace	8.2	6.6	10.4	
1+ or more	8.2	6.7	10.1	
Hematuria (%)	10.5	9.7	11.6	0.363
Medication (%)				
ACEI or ARB	14.4	12.7	16.6	0.091
Anti-platelet agent	21.1	20.6	21.7	0.686
Statin	8.1	9.4	6.5	0.110
No of AntiHTN (%)				0.196
0	53.5	55.4	51.1	
1	28.9	28.5	29.4	
2	10.7	9.4	12.5	
≥3	6.9	7.7	7.0	

GFR: Calculated by CKD-EPI equation, BMI: body mass index, Proteinuria: measured by dipstick test, Anemia: defined in female with hemoglobin less than 12 g/dL and, in male, less than 13 g/dL. Hematuria: RBC≥5/HPF, Anti-platelet agent: aspirin 100 mg, triflusal, sarpogrelate, or clopidogrel, No of AntiHTN: number of antihypertensive medication.

### All-cause Mortality

**Figure 2:**
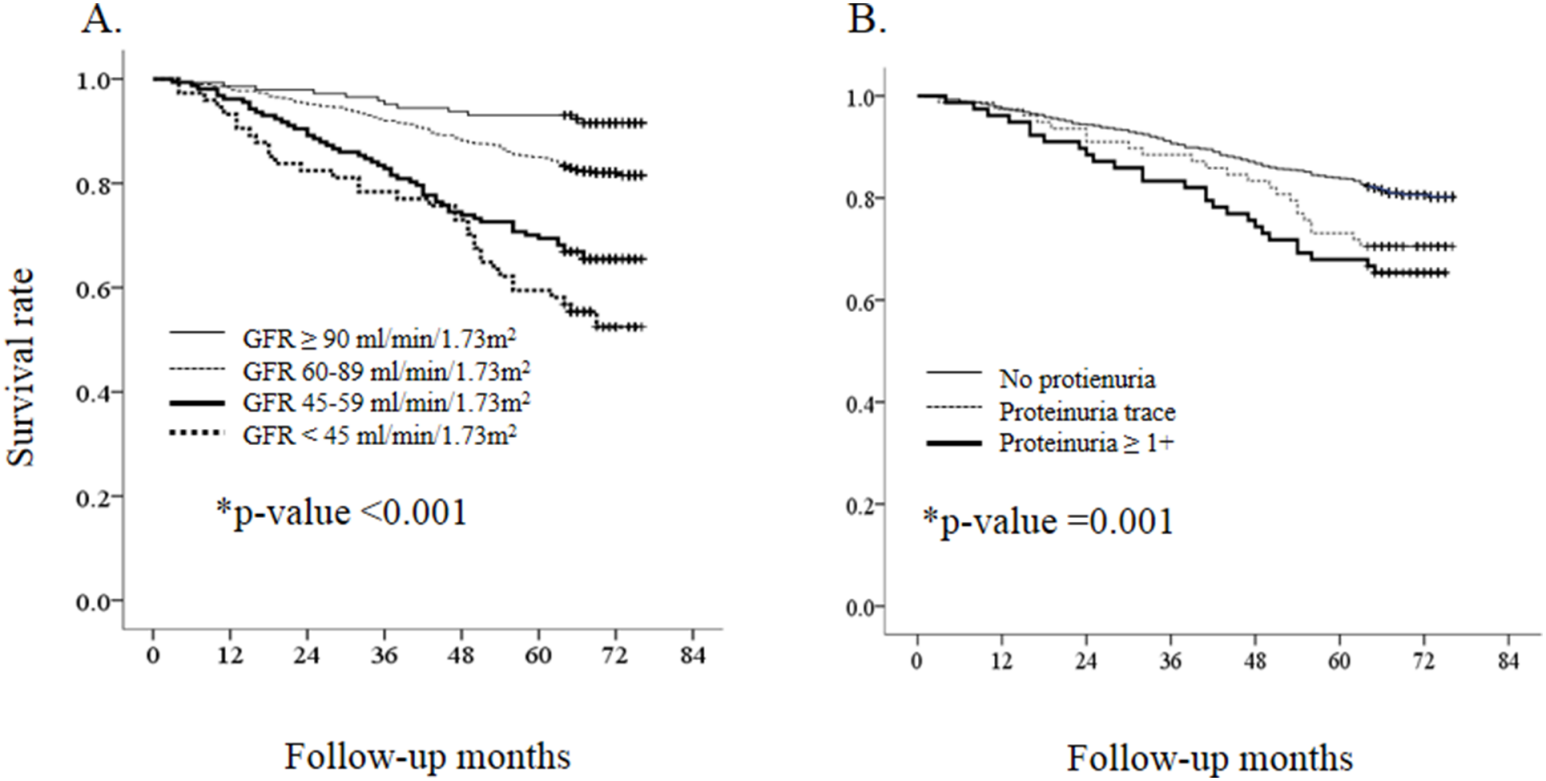
The survival rate according to GFR and proteinuria. A. GFR group for all-caused mortality. B. Proteinuria group for all-caused mortality *p-value by Log-rank test.

The follow-up duration for mortality was 63.4±16.4 months after the baseline visit. Overall, there were 203 cases of ACM and 4.05 deaths/100 person-years (Table 2). The estimated 5-year survival rates were 91.5% in GFR group 1, 89.7% in GFR group 2, 66.9% in GFR group 3, and 52.5% in GFR group 4 (p<0.001; Figure 2). The estimated 5-year survival rates were 92.4% in subjects with no proteinuria, 70.5% in subjects with trace proteinuria, and 65.4% in subjects with proteinuria ≥1+ (p = 0.001; Figure 2). In the estimation of mortality, no interactions were noted between the GFR group and proteinuria group, age group, or gender and between proteinuria group and age group or gender. Subjects with proteinuria of grade ≥1+ had a 1.725-fold higher risk for ACM than those without proteinuria (p = 0.011), and subjects with GFR<45 ml/min/1.73 m^2^ had a 2.357-fold higher risk for ACM than those with GFR ≥90 ml/min/1.73 m^2^ (p = 0.016; Table 3).

**Table 2:** The event rate of all-cause mortality according to basal characteristics.

	N	ACM rate		*p* *
		/100 PY	(95% CI)	
All	949	4.05	3.98–4.11	
Gender				0.005
Male	431	4.92	4.80–5.06	
Female	518	3.34	3.27–3.41	
Age (years)				<0.001
<75	534	1.27	1.25–1.28	
≥75	415	8.48	8.20–8.77	
DM				0.825
no	719	4.10	4.02–4.18	
yes	230	3.90	3.78–4.03	
Hypertension				0.576
no	248	3.81	3.69–3.94	
yes	700	4.14	4.06–4.22	
DBP (mmHg)				0.246
<80	196	5.03	4.82–5.27	
80–89	492	3.67	3.62–3.78	
≥90	259	4.03	3.92–4.15	
SBP (mmHg)				0.707
<130	397	4.30	4.18–4.42	
130–149	350	3.73	3.64–3.83	
≥150	200	4.15	4.02–4.30	
BP (mmHg)				0.895
<130/80	165	4.23	4.04–4.44	
≥130/80	782	4.02	3.95–4.09	
GFR (ml/min/1.73 m^2^)				<0.001
≥90	144	1.47	1.43–1.51	
60–89	574	3.32	3.26–3.38	
45–59	157	7.10	6.74–7.50	
<45	74	10.23	9.36–11.29	
Proteinuria				0.001
none	793	3.61	3.55–3.67	
trace	78	5.79	5.44–6.20	
≥1+	78	7.14	6.63–7.75	

*ACM: all-cause mortality, p-value: calculated by Pearson’s Chi-square test, 100 PY: 100 person-years.

**Table 3:** The significant effect on mortality of proteinuria and GFR.

	HR for ACM*
**Proteinuria group**	
None	1.000 (ref.)
trace	1.235 (0.777–1.963)
≥1+	1.725 (1.134–2.625)
**GFR group**	
≥90	1.000 (ref.)
60–89	1.370 (0.746–2.517)
45–59	1.654 (0.841–3.254)
<45	2.357 (1.170–4.750)

*Cox’s hazard proportional model adjusted with age, gender, habit of smoking, CRP, cholesterol, triglyceride, albumin, platelet, and hemoglobin.

ACM: all-cause mortality, ref.: reference group, HR: Hazard ratio, CI: confidence interval, NC: cannot be calculated.

### GFR Decline and Renal Progression

The follow-up study was conducted 59.4±6.9 months after the baseline visit. Among the 472 subjects who underwent the follow-up examination, 403 were included in the analysis of renal progression. The annual rate of GFR change during the follow-up period was −0.52±2.35 ml/min/1.73 m^2^/year. Compared to participants without DM or lower GFR, those with DM or lower GFR showed a more rapid decline in GFR during the follow-up period (Table 4). The proportion of subjects with renal progression was 11/403 (2.7%). There was no interaction between GFR group and age or DM for renal progression. Negative interactions were found between gender and GFR group (p-interactions = 0.047). Multiple logistic regression analysis revealed that the renal progression rate was 7.315-fold (1.841–29.071) higher in subjects with GFR<60 ml/min/1.73 m^2^ than in those with GFR ≥60 ml/min/1.73 m^2^.

**Table 4:** GFR decline according to baseline characteristics in participants followed up 2^nd^ examination.

	N	GFR decline *	p-value		N	GFR decline *	p-value
		Mean (95% CI)				Mean (95% CI)	
All	403	0.52 (0.31–0.73)		SBP (mmHg)			0.510
Gender			0.237	<130	164	0.42 (0.07–0.76)	
Male	206	0.64 (0.35–0.93)		130–149	158	0.49 (0.16–0.82))	
Female	197	0.15 (0.09–0.69)		≥150	81	0.77 (0.29–1.26)	
Age (years)			0.677	BP (mmHg)			0.068
<75	308	0.47 (0.15–0.78)		<130/80	67	0.08 (***0.44*** ** ***–***0.59)	
≥75	75	0.68 (***0.11*** ** ***–***1.47)		≥130/80	336	0.61 (0.38–0.83)	
DM			<0.001	GFR (ml/min/1.73 m^2^)			0.012
no	304	0.29 (0.05–0.53)		≥90	84	0.29 (***0.40*** **–0.96)	
yes	99	1.21 (0.79–1.63)		60–89	260	0.38 (0.11–0.64)	
HTN			0.084	45–59	41	1.22 (0.33–2.10)	
no	112	0.22 (0.18–0.62)		<45	18	2.88 (1.41–4.35)	
yes	291	0.63 (0.39–0.88)		Proteinuria			0.892
DBP (mmHg)			0.589	none	357	0.53 (0.31–0.75)	
<80	75	0.28 (0.21–0.78)		trace	24	0.56 (***0.30*** ** ***–***1.42)	
80–89	216	0.57 (0.28–0.86)		≥1+	22	0.31 (***0.59*** **–1.21)	
≥90	112	0.57 (0.15–0.99)					

*GFR decline*: GFR decline rate (ml/min/1.73 m^2^/year) estimated by covariate analysis adjusted for age, gender, DM, HTN, GFR, and proteinuria by dipstick test at baseline study.

**The number with bold character indicates GFR increase rate.

## Discussion

In this community-based elderly cohort, the grade of GFR was found to be an independent risk factor for ACM. However, subjects with a GFR of 45–59 ml/min/1.73 m^2^ did not have higher risk for ACM than those with GFR ≥90 ml/min/1.73 m^2^. Proteinuria was an independent risk factor for ACM. The annual rate of GFR decline was 0.52 ml/min/1.73 m^2^. GFR<60 ml/min/1.73 m^2^ was an important risk factor for renal progression.

Studies analyzing the relationships between GFR or proteinuria and outcomes in the elderly have shown variable results depending on the characteristics of the enrolled population. Recent meta-analyses have shown that the HRs of low GFR (<60 ml/min/1.73 m^2^) with ACM [7], [16], [17], CVM[16], and end stage renal disease (ESRD) [7] were similar meanings irrespective of patient age. The studies included all of community populations, high-risk populations for CKD, and CKD populations and showed differences compared to community-based studies. The relationship between serum creatinine level and mortality was most evident in populations with traditional cardiovascular risk factors or renal insufficiency, and not in community-based cohorts [18]. Among patients (age, >55 years) who had documented vascular disease or diabetes with symptoms of end organ damage, the HRs for ACM according to GFR levels increased in proportion to the severity of the traditional risk factors for cardiovascular disease [19]. A study on patients visiting the outpatient clinics showed a less evident relationship between mild-to-moderately decreased GFR and outcomes. O’Hara et al. reported that, compared to elderly patients with GFR ≥60 ml/min/1.73 m^2^, those with a GFR of 50–59 ml/min/1.73 m^2^ did not have an increased risk for death [20]. A prospective cohort study of community-dwelling persons aged ≥65 years showed that GFR was not a risk factor for ACM and CVM [8]. Therefore, a randomly sampled community-based cohort with representativeness is more suitable for determining the effect of changes of GFR with aging on outcomes.

In this respect, the KLoSHA protocol offered several advantages in the evaluation of the relationship between renal parameters and outcomes. The majority of these data were sampled by random selection from non-institutionalized habitants. A prospective study provides higher quality of evidences than those provided by a cross-sectional study. We were able to contact almost all the participants for confirmation of mortality and verified this information against the data maintained by the National Statistical Office of Korea. All Koreans have a unique identifier, which is the primary key to obtain individualized data. Finally, serum creatinine was measured in a single institution throughout the study period and represented as IDMS-traceable value, which was suitable for application of the CKD-EPI equation.

The definition of GFR for the assessment of increased risk of mortality in elderly population can also vary with the nature of the included population. Several prospective community-based cohort studies showed that a GFR value of 60 ml/min/1.73 m^2^ was not a suitable cut-off for the prediction of ACM [8], [9], [20] or CVM [9], [10] among the elderly population. Other studies, including those on community populations, showed no relationship between CVM and GFR in populations of subjects aged ≥65 years [8], [18], [21] or ≥70 years [17]. The other issue is the effect of albuminuria among participants with decreased GFR on mortality. In elderly patients aged ≥60 years, GFR<60 ml/min/1.73 m^2^ estimated by serum creatinine was found to be a risk factor in participants with albuminuria, but not for those without albuminuria [22]. Albuminuria has been reported as a risk factor for ACM and CVM in elderly populations with high risk(s) [11], [16] and in community-based elderly cohorts [11], [23], [24], [25]. For example, in the PREVEND study, albuminuria, but not GFR, was found to be associated with CVM and ACM [11].

A few prospective studies have evaluated the association of age with the decline in estimated GFR with aging [2]. A longitudinal study on an elderly cohort (60–93 years) in Sweden revealed that the GFR estimated by the CKD-EPI equation declined at an annual rate of 1.042 ml/min/1.73 m^2^ in men and 0.970 ml/min/1.73 m^2^ in women [26]; these rates are higher than those noted in this study. Imai et al. reported that the annual decline in GFR using the MDRD equation in an elderly population (60–79 years) followed for 10 years [27] was 0.32–0.39 ml/min/1.73 m^2^ per year, which is similar to that obtained in our study. Ethnic differences in the rate of decline in GFR should be accounted for in a well-designed study. We used the criteria for renal progression used in the KDIGO controversy conferences [15]. Our study revealed that a GFR<60 ml/min/1.73 m^2^ was the most important risk factor of renal progression, as observed in other studies [8], [28].

There are several limitations in the interpretation of this study’s results. The CKD-EPI equation has not been fully validated in elderly Koreans, although our colleagues have reported that the ethnic coefficients of the CKD-EPI equations were close to “1,” on the basis of a study on 131 CKD patients and healthy volunteers [29]. We followed the participants over 5 years but 29.3% of initial population was not followed up at the second examination. The significant percent of dropout rate is an important limitation of this study. Especially, renal progression was evaluated in only subjects who completed follow up examination. Several criteria for renal outcomes have been proposed thus far, and we used the criteria defined at the KDIGO controversies conference [15]. In addition, we evaluated a relatively small numbers of subjects with severely decreased renal function and proteinuria. The results should be carefully interpreted in subjects with eGFR<45 ml/min/1.73 m^2^ or proteinuria. We evaluate the renal outcome on the basis of the difference of serum creatinine at baseline and at the end of the follow up study, and we did not confirm the sustained decline in eGFR during follow up period. Finally, this study is based on a city population, and the generalization of the results for a nationwide population or for all Asians is debatable.

In conclusion, we found that in a community-dwelling elderly Korean population, a decreased GFR<45 ml/min/1.73 m^2^ and proteinuria were independent risk factors for ACM. With aging, the annual rate of decrease in GFR was 0.52 ml/min/1.73 m^2^/year and the baseline GFR was the most important risk factor for renal progression.
